# Sauna use as a novel management approach for cardiovascular health and peripheral arterial disease

**DOI:** 10.3389/fcvm.2025.1537194

**Published:** 2025-03-11

**Authors:** Sergio Sastriques-Dunlop, Santiago Elizondo-Benedetto, Mohamed A. Zayed

**Affiliations:** ^1^ Section of Vascular Surgery, Department of Surgery, Eastern Virginia Medical School, Norfolk, VA, United States; ^2^ Section of Vascular Surgery, Department of Surgery, Washington University School of Medicine, St. Louis, MO, United States; ^3^ Department of Radiology, Washington University School of Medicine, St. Louis, MO, United States; ^4^ Division of Molecular Cell Biology, Washington University School of Medicine, St. Louis, MO, United States; ^5^ Division of Surgical Sciences, Department of Surgery, Washington University School of Medicine, St. Louis, MO, United States; ^6^ Department of Biomedical Engineering, McKelvey School of Engineering, Washington University School of Medicine, St. Louis, MO, United States; ^7^ Department of Surgery, Veterans Affairs St. Louis Health Care System, St. Louis, MO, United States

**Keywords:** peripheral arterial disease, cardiovascular disease, heat therapy, sauna bathing, cardiovascular health, exercise therapy

## Abstract

**Introduction:**

Heat therapy (HT), particularly in the form of whole-body sauna bathing, has emerged as a promising intervention for the management of cardiovascular disease (CVD). Passive HT can induce both local and systemic physiological responses, primarily through repeated thermal stress consisting of short-term passive exposure to high temperatures. Such responses closely parallel the physiological adaptations observed during aerobic exercise. Peripheral arterial disease (PAD) poses significant health challenges, impacting millions of individuals worldwide. Supervised exercise is considered a cornerstone therapy for PAD, yet many patients face significant health-related barriers that complicate its broad implementation.

**Methods:**

We conducted a comprehensive review of the literature to explore the therapeutic implications of various HT practices beyond sauna. The review aimed to evaluate the potential use of these practices as adjunctive management strategies for cardiovascular diseases, particularly in patients with PAD.

**Results:**

Recent studies have demonstrated the potential role of HT in alleviating PAD symptoms, improving functional capacity, and reducing cardiovascular and limb events. HT practices might be beneficial as adjunctive management strategies, in addition to or as alternatives to exercise, for management of cardiovascular diseases.

**Discussion:**

This review highlights the potential benefits, underlying mechanisms of action, challenges, and safety considerations associated with HT. We emphasize the importance of exploring HT as a viable option for patients with cardiovascular conditions, particularly those with PAD, who face barriers to traditional exercise regimens.

## 1 Introduction

Peripheral arterial disease (PAD) is a major circulatory problem characterized by diminished arterial blood flow that predominantly impacts the lower extremities. It carries significant morbidity, mortality, and quality of life impairment. Principally a manifestation of atherosclerosis, the prevalence of PAD increases with age (1, 2). PAD-related functional impairment is associated with increased rates of hospitalization, interventions to attempt limb salvage, and functional disability (1, 2). Current estimates suggest that PAD affects over 230 million individuals worldwide and more than 29% of high-risk individuals in the United States (US), which include individuals over 70 years or with a history of smoking or diabetes mellitus (3). Significantly, the global incidence of PAD has risen by approximately 45% from 2000 to 2015, with greater increases in low- and middle-income countries, although prevalence remains overall higher in high-income countries (3). PAD is linked to most forms of cardiovascular disease (4, 5) such as, dyslipidemia, hypertension, coronary artery disease, and cerebrovascular disease. Lower extremity PAD presents in a spectrum of clinical manifestations, including intermittent claudication, rest pain, tissue loss, and occasionally atypical symptoms linked to ischemia (6). Therefore, medical interventions and risk factor modifications constitute essential elements of evidence-based PAD care. Such strategies are crucial not only for improving cardiovascular morbidity and mortality, but for enhancing limb-related outcomes as well (5, 7).

The American Heart Association/American College of Cardiology (AHA/ACC) guidelines recommend that patients with PAD receive a comprehensive program of guideline-directed medical therapy based on four pillars: statin therapy to reduce hyperlipidemia, antiplatelet therapy, smoking cessation therapy/counseling, and supervised exercise therapy programs (SEPs) (6–9). Guideline-directed medical therapy should also be customized to manage co-morbidities such as diabetes mellitus or hypertension. Patients with lifestyle-limiting claudication despite optimal medical therapy and those with chronic limb-threatening ischemia should be considered for surgical intervention and attempted revascularization. The aim of guideline-directed medical therapy and surgery is to improve patient symptoms, preserve limb function, and enhance quality of life. It is also recommended for all stages of PAD, including post-revascularization (10–12).

While there is strong adherence to pivotal pharmacological therapies such as antiplatelet and statin medications, adherence to non-pharmacological interventions, including smoking cessation and SEP, remains significantly low (7, 13–15). Moreover, such non-pharmacological interventions offered to PAD patients exhibit a highly variable degree of compliance, especially for SEPs and other home exercise programs (9). Consequently, while adherence can be as high as 90% in certain regions of Europe, rates are drastically lower in the US, often ranging from 0 to 10% (7). Noteworthy, there is a strong evidence base to support a central role of structured exercise programs (16), however many PAD patients, particularly those with chronic limb-threatening ischemia, may be unable or unwilling to engage in SEPs or home exercise programs. Because of this, there is an ongoing need for a more viable alternative or adjunctive therapy to alleviate symptoms, improve function, and reduce cardiovascular and limb events especially in individuals who are unable to regularly exercise.

In recent decades, heat therapy (HT) has emerged not only as a tool for relaxation, but also as a novel treatment option for various health concerns. Traditionally, repeated HT can be obtained in the form of whole-body, also known as “sauna bathing,” which has been associated with numerous health benefits (17–22). Similarly, regional or localized body thermotherapies have also demonstrated substantial health benefits (23–26). The therapeutic benefits of HT have been consistently documented in diseases that significantly impact lifespan. For example, neurologic conditions, including stroke, dementia, and psychotic disorders have benefited from HT (27–29). Similarly, patients with lung conditions such as asthma, chronic obstructive pulmonary disease, and pulmonary hypertension have observed functional improvement, further contributing to lifespan extension (17, 30–32).

In terms of cardiovascular health, numerous preclinical and clinical studies suggest that regular or frequent exposure to passive HT reduces the risk of cardiovascular diseases. For example, studies have demonstrated that HT can improve conditions such as hypertension, venous thromboembolism, heart failure, and overall cardiovascular disease (CVD) mortality (19, 33, 34). Similarly, other forms of whole-body HT, such as Waon therapy, have gained popularity for their role in peripheral vascular disease (35). Practices that involve localized body thermotherapy have also shown a positive impact on cardiovascular health (23–26). HT replicates the physiological benefits of aerobic exercise, suggesting its potential utility in the management of CVD and PAD (18, 35), particularly for patients who are unable to engage in physical activity. An important question that remains is whether these HT practices could be strategically implemented to enhance the management of PAD.

This review focuses on the preclinical, epidemiological, and interventional evidence indicating implementation of various HT practices and their potential roles in the management of PAD and CVD. Furthermore, our comprehensive review explores the mechanistic pathways that could potentially influence the effectiveness of passive HT on cardiovascular health outcomes. Given the plethora of HT practices that have been tested and are commercially available, we specifically concentrated on the clinical evidence associated with sauna bathing and the areas currently undergoing active research.

## 2 Whole-body heat therapy: Finnish-style sauna and Waon therapy

Most studies on HT have focused on the use of whole-body Finnish-style sauna bathing. Although HT practices can vary by heat source, temperature, relative humidity, and usage duration (Table 1), the traditional Finnish-style sauna bathing is usually the model of reference. Sauna bathing is a form of passive HT characterized by dry air and the exposure to high temperature for a brief period. The recommended temperature ranges from 80°C to 100°C, while the relative humidity ranges from 10%–20% (36). The heating source is composed of hot rocks (including wood-fired, gas and electric models), and the sauna is usually made of wood with wooden benches well above the floor upon which bathers sit (37). Typical sauna sessions consist of short stays in the sauna room ranging between 5 and 20 min. Sauna sessions can be interspersed with cooling-off periods (swim, shower, or room temperature acclimation). A typical Finnish-style sauna bath occurs at least once per week, with the average habitual frequency being 2–4 times per week (17, 19, 32, 38).

**Table 1:** Heat therapies (HT) overview.

Heat therapies	Whole-body heat therapy			Localized heat therapy		Combined
	Finnish-style sauna bathing	Waon therapy	Hot water immersion	Pulsed shortwave diathermy (PSD)	Heated water	Far infrared (FIR)
Description	Dry air in a wood paneled room	Infrared heat in a closed chamber	Head out water immersion in a hot tub	Deep heat within body tissues	Water-circulating or spa bathing	Lamps or heating elements that emit FIR
Heat source	Sauna heater: • Wood-fired • Gas • Electric	Infrared heat Warmed blankets (keep temp)	Heated water Blankets and water bottles (keep temp)	Electromagnetic radiation	Heat trousers Hot water immersion	Thermal radiation • Incandescent bulbs • Ceramic or metallic
Temp °C (°F)	80–100 (158–212)	60 (140)	40 (104)	Raises tissue temperature by 3.9 (39)	48 (118)	45–68 (113–150)
Duration	5–20 min	15 min (inside) 30 min (outside)	30 min (inside) 90 min (total)	Variable	Variable	Variable

Waon therapy, another prevalent form of dry sauna practice, involves using an evenly heated chamber to envelop the entire body in soothing warmth (31, 39). In Japanese, the term “Waon” translates to “comfortable warmth that refreshes the mind and body” (40). This whole-body HT, as outlined in Table 1, consists of a two-step process: first, a 15-minute session of infrared heat exposure in a sauna heated to approximately 60°C, raising the core body temperature by about 1.0°C–1.2°C; second, participants lie supine outside the sauna while covered in warm blankets to maintain this temperature for an additional 30 min (18, 40). Widely available, this popular sauna therapy improves hemodynamics and cardiac function without severe adverse effects (41). Other less popular whole-body HTs include steam rooms (100% humidity) and whole-body hyperthermia, a method which uses radiation, convection, or conduction to produce heat (17).

Another effective form of whole-body HT can be achieved through repeated hot water immersion, also known as hot “head-out” water immersion (42–46). In this method, subjects are immersed up to the shoulders or sternum (typically with arms out) in a hot tub maintained at 40°C for approximately 30 min, increasing body temperature by 0.6–0.8°C (Table 1). Following this initial phase, subjects may sit on a bench with water reaching waist level to maintain the elevated temperature for an additional 30–60 min, with a maximum total exposure of 90 min (46). This approach has shown to be the most effective passive hyperthermia method for inducing classical markers of heat acclimation and increasing heat-related proteins (45). Alternatively, subjects can lie on a lounge chair, wrapped in warm blankets with hot water bottles placed on the abdomen and thighs for at least 30 min (43).

## 3 Localized and combined heat therapy

Local/regional-body thermotherapy uses direct contact with a heated liquid (such as water or wax), hot blankets or suits, heating coils, or specialized lamps that emit infrared-A radiation to target a specific region of the body (17).

Despite only targeting a specific area, selective immersion of the legs in hot water can still impact circulatory parameters, including cardiac output, stroke volume, and heart rate (47). To illustrate, localized HT applied with customized water-circulating trousers can increase popliteal artery blood flow, lower blood pressure and reduce vasoconstriction (48). Using this modality, specialized tube-lined trousers that are filled with heated water at 48°C is applied for 90 min, 3 times per week (Table 1). Compared to a sham group, such intervention improved the perceived physical function in patients with PAD (24). Similarly, the use of pulsed shortwave diathermy, another form of repeated exposure to heat stress that promotes deep tissue heating, demonstrated beneficial heat-induced mitochondrial adaptations in human skeletal muscle (24, 49).

Infrared heaters emit thermal radiation, which heats the body directly. They operate at lower temperatures than traditional saunas, at 45°C–65.5°C. Infrared heaters emit either near or far wavelengths (typically from 0.75 to 1000 μm), which permit their use as a “combined” heat source (Table 1) for either whole or localized-body therapies. This modality uses incandescent bulbs to produce thermal radiation of varying wavelengths, ranging from near-infrared wavelengths (0.75–1.5 μm) to middle-infrared (1.5–5.6 μm) wavelengths. Far infrared (FIR) heaters use ceramic or metallic heating elements that emit energy in the far-infrared range (5.6–1000 μm), typically at wavelengths of approximately 10 μm (50). A study conducted in Taiwan evaluated the application of FIR therapy in the upper extremities and demonstrated significantly improved hemodialysis access flow and unassisted patency (51).

## 4 Physiologic responses to heat stress and proposed mechanisms of action

**Figure 1:**
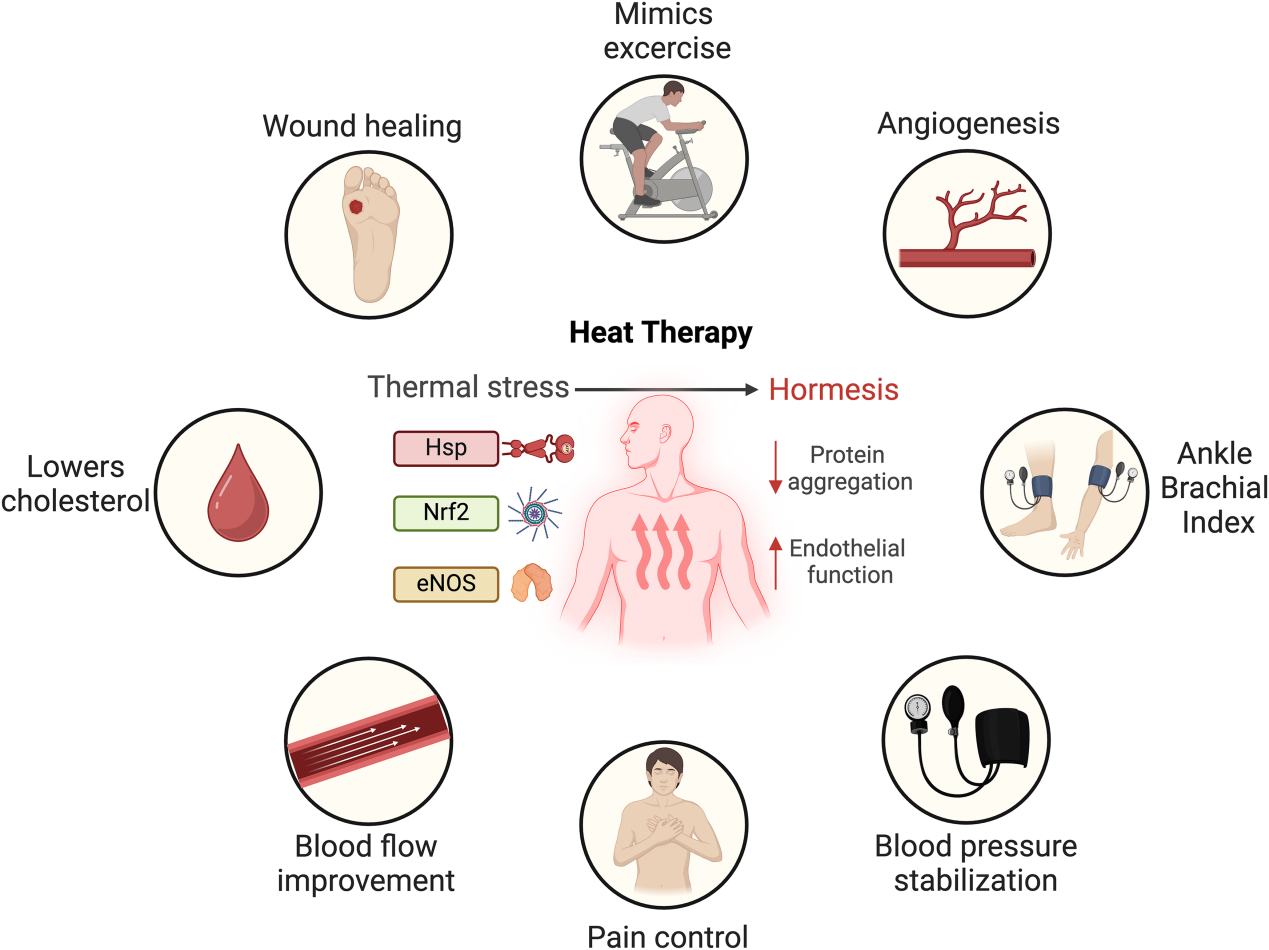
Mechanistic response to thermal stress and proposed cardiovascular benefits of heat therapy. Heat shock proteins (Hsp), nuclear factor erythroid 2-related (Nrf2) and endothelial nitric oxide synthase (eNOS). Created in BioRender. Arif, B. (2025) https://BioRender.com/v00n110, licensed under Academic License.

When the body is exposed to high temperatures, it responds with a rapid physiological reaction primarily impacting the skin and cardiovascular systems. Initially, the skin temperature rises to approximately 40°C, followed by a gradual increase in core body temperature (36, 52, 53). This thermal stress (Figure 1) induces an increase in cardiac output by up to 60%–70%, with elevated heart rate and a stable stroke volume (17, 52). Simultaneously, the body's circulatory system redistributes approximately 50%–70% of its blood flow from the core to the skin, facilitating sweating and driving fluid losses (17, 19, 29, 54). Repeated sauna sessions help the body adapt to heat, improving its ability to handle subsequent exposures. This is attributed to a biological phenomenon known as hormesis, wherein exposure to a mild stressor elicits a compensatory defense response (17, 55) (Figure 1). This response activates a variety of protective mechanisms that repair cellular damage and provide protection against more severe stressors (17, 19, 38, 56).

The physiological responses to sauna bathing bear a striking resemblance to those experienced during moderate to vigorous aerobic exercise (21). For example, passive HT through whole-body water immersion promotes vasodilation and concurrently impacts arterial stiffness, wall thickness, and blood pressure (46, 55). Although both whole-body and local heating strategies notably impact blood flow, it is important to highlight that only local heating significantly enhances muscle blood flow, whereas whole-body heating predominantly increases skin blood flow (57).

On a molecular level, heat stress activates a series of mechanisms that stimulate endogenous antioxidant, repair, and degradation processes. One such mechanism is the increased expression of heat shock proteins (HSPs), a highly conserved family of proteins present in all cells (58). Passive HT has been demonstrated to significantly boost HSP expression in circulating leukocytes (59), subcutaneous adipose tissue (60) and notably, skeletal muscle tissue in humans (49, 61). These proteins play a critical role in protecting cells from the harmful effects of heat and other stressors (58). HSPs also play vital roles under normal conditions, contributing to immune function, cell signaling, cell-cycle regulation, and proteostasis. By repairing damaged proteins, HSPs prevent protein disorder and aggregation, a common feature in age-related diseases (56, 62–64). Additionally, HSPs have been found to positively influence cardiovascular function by modulating nitric oxide pathways, and mitigating chronic oxidative stress and vascular inflammation (58).

Another key molecular player in the heat stress response is nuclear factor erythroid 2–related factor 2 (Nrf2), a regulator of the cellular antioxidant response. Upon activation by heat stress, Nrf2 translocates from the cytoplasm to the nucleus, orchestrating the regulation of a vast network of genes involved in cytoprotective, antioxidant, and anti-inflammatory functions (25, 26, 65). Specifically, heat-activated Nrf2 has been shown to suppress pro-inflammatory molecules involved in CVD, such as E-selectin, vascular cell adhesion molecule-1, and intercellular adhesion molecule-1, through the upregulation of HSP heme oxygenase-1 (17, 26). In summary, while heat exposure induces a robust cellular stress response, causing upregulation of protective proteins and pathways, this does not necessarily result in cellular damage. Rather, the repetitive stress responses induced by HT may offer protective benefits, particularly in the context of cardiovascular disease (58).

In conditions such as congestive heart failure, coronary artery disease, and PAD, sauna bathing can ameliorate myocardial perfusion abnormalities (18, 22). As seen in Figure 1, nitric oxide, produced by endothelial nitric oxide synthase (eNOS), is a main playmaker during thermal stress. Patients with CVD often exhibit reduced nitric oxide bioavailability (66). Repeated heat exposure increases cardiac output and the shear stress of the vessel wall, ultimately leading to enhanced eNOS expression in the arterial endothelium (67, 68). This results in eNOS-dependent mobilization of endothelial progenitor cells and enhanced angiogenesis (35, 39, 40, 69, 70). Furthermore, FIR radiation has been shown to inhibit the tumor necrosis factor -*α*-mediated expression of the adhesion molecules E-selectin, vascular cell adhesion molecule-1, intercellular adhesion molecule-1, as well as the downregulation of chemo-attractants monocyte chemo-attractant protein-1 (MCP-1) and interleukin-8 (IL-8), all of which enhance endothelial function (31).

## 5 Potential benefits of sauna for management of CVD and PAD

A growing body of evidence suggests that beyond its use for pleasure and relaxation, sauna bathing transcends these conventional roles, providing potential health benefits (Figure 1). The potential benefits of passive HT, particularly sauna bathing, in the management of CVD and PAD are supported by clinical evidence and are becoming increasingly recognized (17, 19, 21, 38, 54). Heat exposure elicits protective responses that promote cardiovascular health, mirroring the beneficial adaptations associated with exercise training (55). In a randomized controlled trial (RCT) (21), the combination of sauna exposure and exercise was demonstrated to synergistically enhance cardiorespiratory fitness, resulting in greater reductions in systolic and diastolic blood pressure as well as total cholesterol levels, compared to either exercise or sauna alone (21, 71). For sedentary populations, such as individuals with disabilities, wheelchair users, those on non-weightbearing status or the elderly, performing regular exercise with its associated anti-inflammatory benefits may not be feasible. Therefore, manipulating body temperature could serve as an alternative to potentially mitigate cardiovascular events. In this context, sauna bathing emerges as a potential therapeutic option that might be effective and carries low risk.

In the context of PAD, primarily associative data from studies utilizing Waon therapy have shown significant decreases in pain scores and increases in 6-minute walk distance, ankle-brachial index, and blood flow as assessed by laser doppler perfusion imaging (35). However, a RCT with a larger and more diverse group of PAD patients is crucial to confirm the efficacy of Waon therapy. Moreover, early study findings demonstrated that regular sauna use may help induce new collateral vessel formation as visualized on angiography—suggesting that such therapies may promote angiogenesis and formation of new collaterals (35). In some cases, ischemic ulcers have been reported to have markedly improved healing without the need for invasive therapy (25, 26, 31, 35, 39, 69, 70).

Operatively, the success of peripheral revascularization, whether open or endovascular, largely depends on adequate outflow, which is an independent risk factor for patency. Individuals with PAD frequently suffer from both macro- and microvascular disease (11, 12). In this regard, repeated HT may improve outflow microvasculature which could improve healing and improved outflow following revascularization (Figure 1). Patients with end-stage PAD without revascularization options may benefit even more from this therapy, since they have limited revascularization options for limb salvage. In Japan, patients frequently continue to undergo Waon therapy in the outpatient clinic at least twice weekly after peripheral revascularization (39). However, RCT with systematic follow-up studies are essential to draw definitive conclusions regarding the long-term benefits and outcomes of this therapy.

Most patients with CVD also suffer from end-stage renal disease, necessitating vascular access, which is crucial for delivering essential dialysis treatment. Unfortunately, arteriovenous fistulas or grafts can be prone to complications, such as failure to mature or thrombosis, making them a significant cause of morbidity leading to frequent hospitalizations and interventions in the US (72). Furthermore, there are no approved interventions or guidelines for the prevention of arteriovenous access failure, other than ultrasound routine follow up. In Taiwan, a RCT showed that FIR therapy helps prevent arteriovenous access failure and has gained widespread acceptance as a therapeutic approach (73). Hemodialysis centers worldwide are increasingly embracing this innovative treatment option to enhance maturation rates and sustain access patency for improved patient outcomes (26, 73). Further research and evaluation of guidelines are necessary to tailor the applicability of this therapy in the US, as well as to identify and address potential complications or limitations to this technique.

## 6 Safety and tolerability

Although sauna is generally well tolerated and safe, especially in healthy individuals, there are some contraindications to consider. According to a review of cases done by Luurila et al., alcohol consumption combined with sauna use can significantly increase sudden death (74). The researchers also recommended that “natural contraindications,” such as infectious diseases, acute chest pain, unstable angina pectoris, decompensated heart failure, and tight aortic stenosis should be considered as contraindications. Although these suggestions are logical and anticipate precautions to prevent complications, further evidence is necessary to definitively establish a correlation. Prospective studies are required to elucidate the correlation and risk associated with sauna use in such populations.

Importantly, antihypertensive medication use immediately before bathing is also not advisable as it may predispose the individual to orthostatic hypotension (75). Concerns about male fertility have been raised due to scrotal hyperthermia and altered spermatogenesis. A small study indicated decreased sperm count and motility; however, these effects were reversible within 6 months of discontinuing sauna use (76). On another front, some central nervous system birth defects, including anencephaly and spina bifida, have been associated with extreme heat exposure during pregnancy (77). Paradoxically, in Finland, where most women engage in weekly sauna bathing throughout pregnancy, the incidence of anencephaly is the lowest globally (78). Also, observational studies in both Finland and the US have failed to establish links between sauna use and a higher incidence of cardiovascular or neurological malformations, the most prevalent type of birth defect (75). Lastly, severe adverse effects may result from accidents, such as burns from the heater and drowning during a cool-off swim, with alcohol often contributing to these incidents (75).

## 7 Discussion

In summary, the current body of evidence suggests that passive HT, particularly in the form of sauna bathing, holds promise as a therapeutic intervention for PAD and offers a low-risk and potentially effective option for treatment. The physiological and molecular responses to heat stress mimic those of moderate to vigorous aerobic exercise, potentially alleviating symptoms, improving functional status, and reducing cardiovascular and limb events in PAD patients (Figure 1). However, it is important to note that all HT practices discussed in this review are not currently approved by the US Food and Drug Administration (FDA). The development of HT as potential treatments often relies on preliminary research, highlighting the necessity for further studies to elucidate the mechanisms driving its positive effects. Although our review incorporates a significant number of RCTs over associative studies, it is evident that the scientific rigor of the existing research remains in need of improvement. Moreover, the limited number of high-quality RCTs with large sample sizes and standardized multi-center approaches poses a challenge for conducting a thorough systematic review of the existing literature. Future studies should aim to address the current gaps in the evidence, particularly concerning the long-term effects of heat therapy and its efficacy in diverse patient populations. Finally, it is essential to develop and implement a standard and practical protocol for the application of HT in managing PAD. Establishing consensus on these optimal procedures will optimize its use and potentially enhance patient outcomes.
